# Assessing the role of CT imaging in identifying candidates for neoadjuvant chemotherapy in right colon cancer: a critical analysis

**DOI:** 10.1007/s00384-026-05103-z

**Published:** 2026-02-16

**Authors:** João Leão Lopes, Ana Sofia S. Soares, Beatriz Mendes, Elisa Paoluzzi Tomada, Miguel Cunha, Laura Melina Fernandez, Edgar Amorim, José Azevedo, Amjad Parvaiz

**Affiliations:** 1 Faculty of Medicine, University of Lisbon, Lisbon, Portugal; 2 Surgery Department, Colorectal Surgery Group, Algarve Local Health Unit, Portimão, Portugal; 3 Department of Biomedical Sciences, Humanitas University, Milan, Italy; 4 Colorectal Surgery, IRCCS Humanitas Research Hospital, Milan, Italy; 5 Faculty of Medicine and Biomedical Sciences (FMCB), University of Algarve, Faro, Portugal; 6 Digestive Unit, Champalimaud Clinical Center, Champalimaud Foundation, Lisbon, Portugal; 7 Faculty of Science and Health, University of Portsmouth, Portsmouth, UK

**Keywords:** Right Colon Cancer, Computed Tomography, Diagnostic Accuracy, Neoadjuvant Chemotherapy, Right Hemicolectomy

## Abstract

**Background and purpose:**

Standard treatment for localized right colon cancer is radical surgery, followed by adjuvant chemotherapy for stage III or intermediate MSS and high-risk stage II tumours. Recent studies suggest a benefit from neoadjuvant chemotherapy (NAC), particularly for T4b and/or N + tumours. Patient selection for NAC relies on CT-based clinical staging, but the accuracy of CT in detecting high-risk features is variable, raising concerns about potential overtreatment. The study aims to demonstrate the accuracy of CT staging of the right colon with the purpose of indicating neoadjuvant CT.

**Methods:**

Patients undergoing curative right hemicolectomy between 2013 and 2023 at two Portuguese institutions were included. All had preoperative CT; those receiving NAC were excluded. Sensitivity, specificity, positive predictive value, and negative predictive value of CT in identifying T4b and N + tumours were calculated by comparing clinical (cTNM) and pathological (pTNM) staging.

**Results:**

Among 165 patients (48% male, mean age 70.5 years), CT showed low sensitivity (26%) but high specificity (91%) for pT4b tumours, with a tendency toward understaging. For nodal disease, sensitivity was 87% and specificity 41%. Only 57% of cT4b and/or cN + cases confirmed at least one unfavorable pathological factor, implying potential overtreatment in 43% of patients if NAC were applied solely based on CT findings.

**Conclusion:**

CT remains the standard for clinical staging but demonstrates limited accuracy in identifying high-risk right colon cancers. NAC decisions should integrate additional criteria beyond CT findings to avoid overtreatment.

## Introduction

According to current European Society for Medical Oncology (ESMO) guidelines, the standard treatment for localized colon cancer consists of radical surgical resection, followed by adjuvant chemotherapy in stage III disease or high-risk stage II disease. However, the most recent National Comprehensive Cancer Network (NCCN) guidelines also recommend neoadjuvant chemotherapy (NAC) in patients with adverse prognostic factors, namely T4b stage and/or locoregional nodal involvement [1, 2].

Given that the major prognostic determinant in localized colon cancer is the risk of distant metastasis [3], early systemic control through NAC could improve long-term outcomes. This is particularly relevant in locally advanced, but resectable tumours, which show recurrence rates of 20–30%. When treated with up-front surgery, these tumours present a 3-year DFS of only 53%, compared with 87% in lower-risk tumours [4–9].

The phase III FOxTROT (Fluoropyrimidine Oxaliplatin and Targeted Receptor Pre-Operative Therapy) trial provided robust evidence supporting NAC in locally advanced but resectable (T4 and high risk T3, Nx and M0) colon cancer [10]. Patients randomized to preoperative oxaliplatin-5FU followed by surgery achieved a 28% reduction in 2-year recurrence, higher R0 resection rates (94% vs. 89%), and significant T and N downstaging compared with those undergoing upfront surgery [4, 5, 10]. Consequently, NCCN guidelines now endorse NAC with CAPOX or FOLFOX in cT4b Nx or cTx N + cases, followed by curative surgery and adjuvant chemotherapy.

There is significant biological and clinical heterogeneity within T4 disease. T4a tumours, are characterized by invasion of the visceral peritoneum and are associated with a considerably higher 5-year survival rate (approximately 80%) compared with T4b tumours, which directly invade or adhere to adjacent organs or structures [2]. In right-sided colon cancers, given that the posterior wall is retroperitoneal and lacks peritoneal coverage, tumours arising from the posterior wall of the right colon with invasion of the retroperitoneal fascia – a feature associated with a high incidence of synchronous and metachronous distant metastasis—are classified as T4b [2, 11]. Furthermore, since bowel wall invasion is frequently misclassified on preoperative CT, high-risk T3 tumours (defined by extramural invasion ≥ 5 mm beyond the muscularis propria) with suspected retroperitonal breach may be managed as biologically similar to T4b disease in contemporary NAC algorithms [2, 12, 13].

Accurate preoperative staging is therefore essential for identifying suitable candidates for NAC. However, FOxTROT also revealed substantial limitations of computed tomography (CT)-based staging: only 47% of pT4 tumours were classified as cT4, and 50% of cT4 cases were actually pT3. Similarly, 44% of those defined as cN + at CT imaging tumours proved pathologically node-negative [4]. These findings, supported by other studies, underscore CT’s variable sensitivity and specificity in T and N assessment [14]. Overstaging may lead to unnecessary chemotherapy, with risks of toxicity, surgical delay, and perioperative morbidity [4, 5, 8, 15].

Despite these concerns, previous evaluations of CT accuracy have rarely differentiated between right- and left-sided colon cancers, which differ markedly in epidemiology, molecular profile, metastatic patterns, and outcomes [16–22].

Against this background, the present study aims to evaluate the diagnostic performance of CT in staging locally advanced right-sided colon cancers and to estimate the risk of overtreatment and undertreatment if NAC were to be applied based on CT findings.

### Methods

A national, multicentre, retrospective study was conducted using prospectively maintained databases from two Portuguese institutions, *Champalimaud Clinical Centre * and *Hospital de Portimão*. The study encompassed all patients who underwent elective curative-intent right hemicolectomy for right-sided colon cancer between 2013 and 2023. Eligible patients had an available preoperative CT for staging, histologically confirmed malignancy (defined by ICD-O-3), no NAC, and underwent R0 or R1 resection (with R1 defined as the presence of tumour ≤ 1 mm from the retroperitoneal margin). Patients who had received NAC, underwent R2 resection, or had incomplete clinical (cT) or pathological staging were excluded.

Collected variables included demographic data (age, sex and institution), clinical staging (tumour location and cTNM classification), and histopathological features (pTNM, tumour histology, lymph node yield, and resection type). Nodal status was dichotomized as positive or negative based on the presence of nodal metastases. Pathological staging followed the same TNM system. At both institutions, CT imaging was performed with a 1-mm slice thickness using a standard multiphasic abdominal triple‑phase protocol, including non‑contrast, arterial (approximately 25–35 s), and portal venous (approximately 70–80 s) phases following intravenous injection of iodinated contrast. Pathological examination at both institutions followed standard protocols, performed by gastrointestinal (GI) dedicated pathologists trained in colorectal cancer staging. In both institutions, the retroperitoneal margin was inked, and no specialized fat-clearing techniques for lymph node retrieval or routine immunohistochemistry for high-risk feature detection were used. All cases were reviewed at each institution by a team that included GI pathologists and radiologists with > 5 years of specialized experience and discussed at multidisciplinary tumour boards. Surgical interventions were also standardised and performed by dedicated colorectal surgery teams.

The primary endpoint was the diagnostic performance of CT in identifying adverse pathological features (T4b or N +). Sensitivity, specificity, positive predictive value, negative predictive value, and overall accuracy were calculated using pathological staging as the reference standard.

The secondary endpoints were:

1. the proportion of tumours staged as high risk (cT4b and/or cN +) that did not meet pathological criteria for neoadjuvant chemotherapy (potential overtreatment), and
2. the proportion of clinically low-risk tumours (cT1–4a N0) that were proved pathologically eligible for adjuvant chemotherapy (potential undertreatment).

All statistical analyses were performed using Microsoft Excel (Microsoft Corporation, Redmond, WA, USA).

## Results

**Fig. 1:**
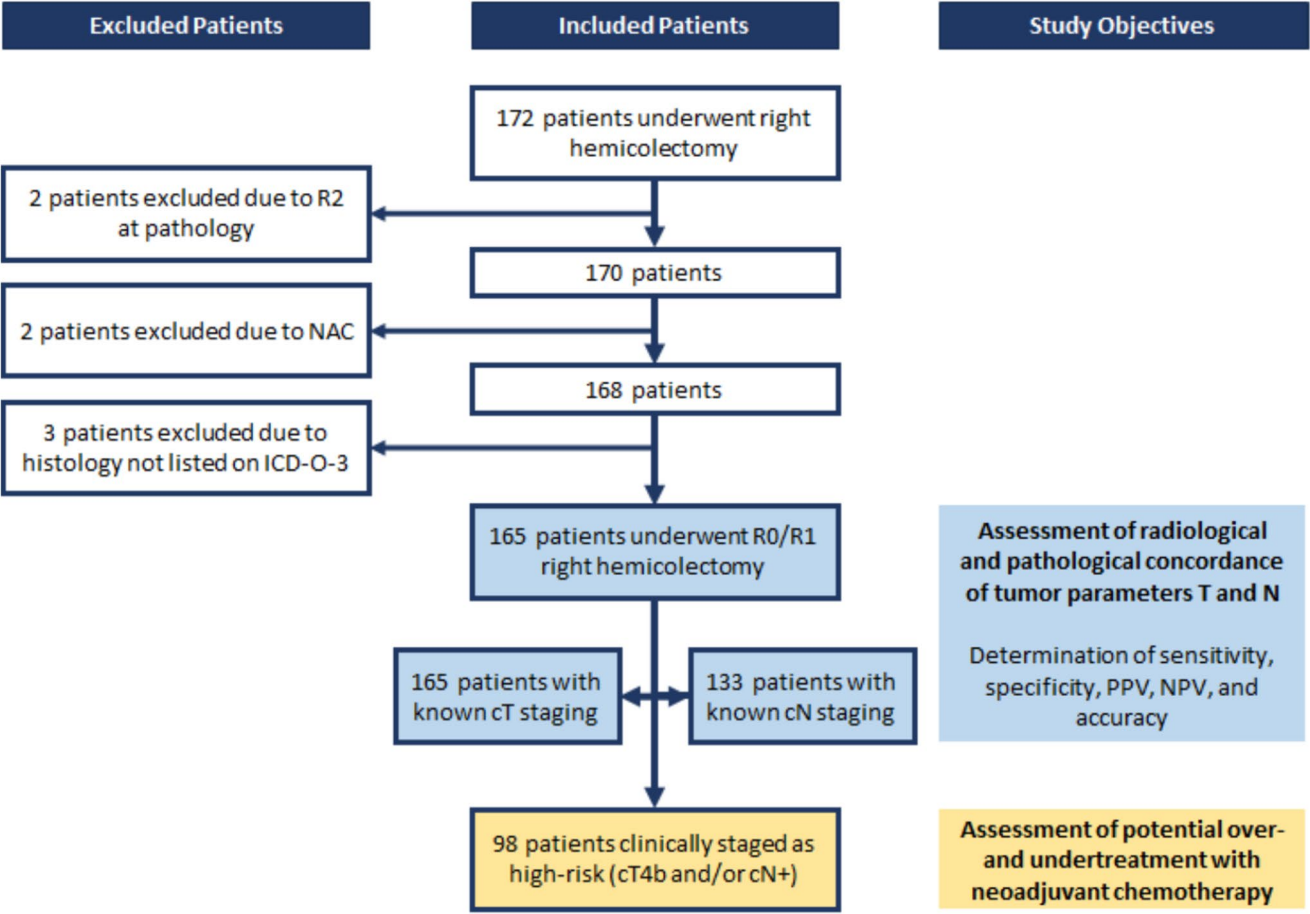
Flowchart illustrating the study selection process

Out of 172 patients with data on clinical and pathological staging, 2 were excluded due to R2 at pathology, 2 were excluded because they underwent NAC treatment and 3 were excluded because of histology not listed on ICD-O-3. A total of 165 patients were included, comprising 80 males (48%) and 85 females (52%), with a mean age of 70.8 years (range, 37–92 years). Overall, 81 (49%) patients presented at least one adverse pathological factor, including 7 (4%) with pT4b and 77 (47%) with pN + as seen in Fig. 1.

Only 133 patients (81%) had known clinical N status (cN0 or cN +); therefore, the comparative analysis of preoperative CT staging and histopathological findings was performed separately for T and N parameters (Table 1).

**Table 1:** Patients’ demographics and clinical and histologic tumour characteristics

Sex	
Male	80 (48%)
Female	85 (52%)
Age (years)	
Mean; range	70,8; (37–92)
Institution	
C.C. Champalimaud	72 (44%)
H. Portimão	93 (56%)
Tumour Location	
Cecum	65 (39%)
Ascending Colon	52 (32%)
Hepatic Flexure	29 (18%)
Transverse Colon	19 (11%)
Lymph Nodes (LN) Excision	
No. of harvested LNs (mean; SD)	39 (22,9)
No. of positive LNs (mean; SD)	5 (4,8)
Resection Type	
R0	160 (97%)
R1	5 (3%)
T Clinical Stage (cT, n = 165)	
cT1	7 (4%)
cT2	41 (25%)
cT3	96 (58%)
cT4a	12 (7%)
cT4b	9 (6%)
N Clinical Stage (cN, n = 133)	
cN0	40 (30%)
cN +	93 (70%)
cNx	32
M Clinical Stage (cM, n = 165)	
cM0	151 (91%)
cM1	14 (9%)
T Pathological Stage (pT, n = 165)	
pT1	7 (4%)
pT2	25 (15%)
pT3	103 (63%)
pT4a	23 (14%)
pT4b	7 (4%)
N Pathological Stage (pN, n = 165 | 133)	
pN0	88 (53%) | 68 (51%)
pN +	77 (47%) | 65 (49%)
Tumour Histology (n = 165)	
Adenocarcinoma	144 (87%)
Mucinous carcinoma	17 (10%)
Signet-ring cell carcinoma	4 (3%)

### Clinical (CT) and pathological staging concordance – T stage

Clinical staging identified 58% tumours as cT3, 7% as cT4a and 6% as cT4b, whereas pathological assessment showed 62% pT3, 14% pT4a and 4% pT4b (Table 2.).

**Table 2:**
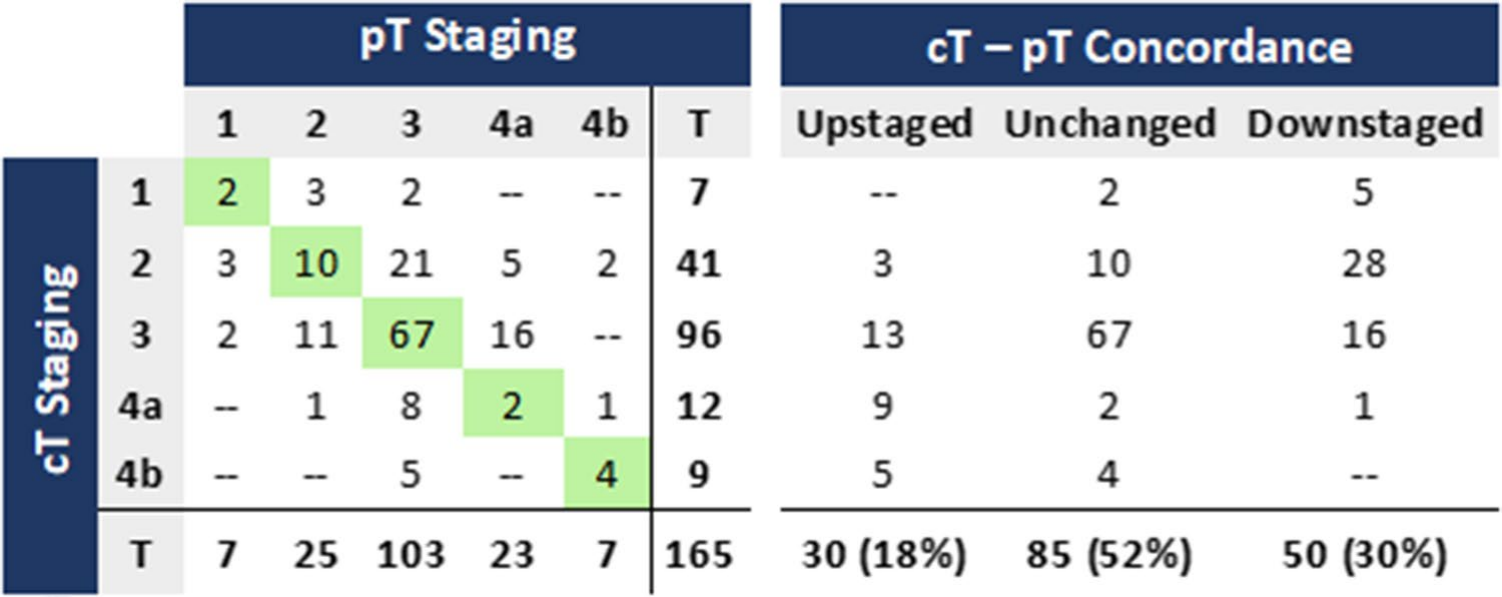
Concordance between clinical and pathological T staging

CT understaged 50 of 165 cases (30%) and overstaged 30 (18%), with an overall concordance of 52% (85/165). Among tumours staged clinically as cT4b, 56% (5/9) were found to be pT3, and 44% (4/9) pT4b. Additionally, 3 of 7 pT4b tumours (43%) were clinically understaged.

For the detection of T4b tumours, CT sensitivity was 57%, specificity was 97%, positive predictive value was 44%, negative predictive value was 98%, and accuracy was 95% (Table 3.).

**Table 3:**
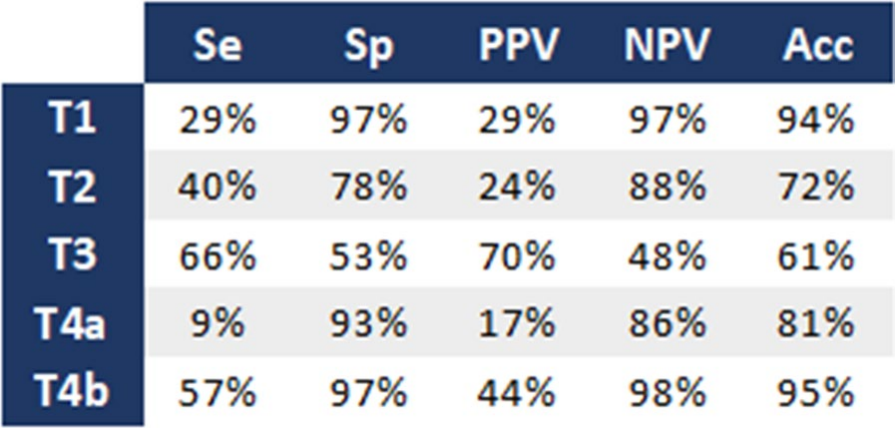
Performance of CT in clinical T staging, compared with histopathology

*Se* sensitivity, *Sp.* Specificity, *PPV* positive predictive value, *NPV* negative predictive value, *Acc.* accuracy

### Clinical (CT) and pathological staging concordance – N stage

Among the 133 patients with available cN status, CT suggested locoregional spread in 70% (93/133). Pathological staging showed 68 (51%) pN0, and 65 (49%) pN + (Table 4.).

**Table 4:**
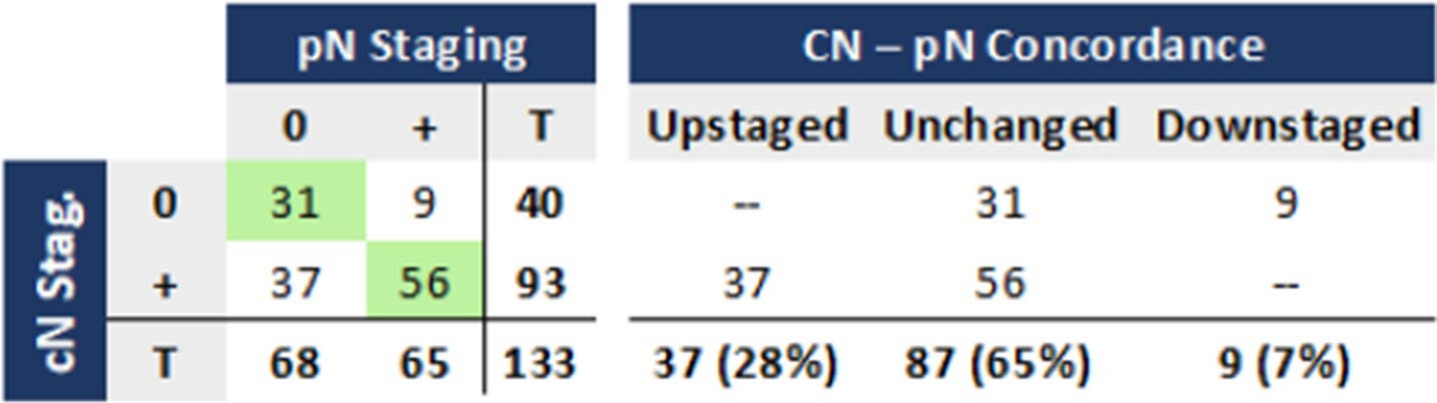
Concordance between clinical and pathological N staging

CT overstaged nodal status in 37 of 93 cN + tumours (40%) and understaged in 9 of 40 cN0 tumours (23%), yielding an overall concordance of 65% (87/133). For detection of nodal metastases (N + disease), CT demonstrated a sensitivity of 86%, specificity of 46%, positive predictive value of 60%, negative predictive value of 78%, and overall accuracy of 65% (Table 5.).

**Table 5:**
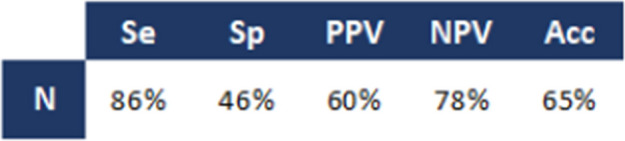
Performance of CT in clinical N staging, compared with histopathology

*Se.* sensitivity, *Sp.* Specificity, *PPV* positive predictive value, *NPV* negative predictive value, *Acc.* accuracy

### Assessment of potential over- and undertreatment with neoadjuvant chemotherapy

Of the 165 patients, 98 (59%) were clinically staged as high-risk (9 cT4b, 93 cN +), thus meeting criteria for neoadjuvant chemotherapy (NAC). CT identified a single high-risk feature in 94 patients (96%) and both T4b and N + features in 4 patients (4%) (Table 6.).

**Table 6:**
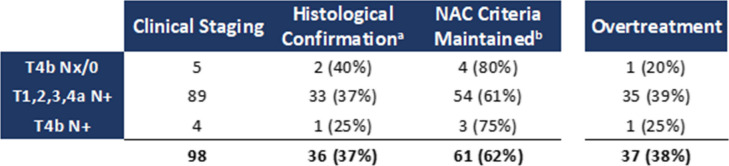
Comparison of clinical and pathological staging of tumours preoperatively defined as eligible for neoadjuvant chemotherapy according to NCCN/FOxTROT study criteria

A – Cases with histopathological confirmation of preoperative staging defined by CT. b – Cases in which, regardless of histopathological confirmation of preoperative staging, criteria for neoadjuvant chemotherapy eligibility are maintained

Pathological assessment revealed discordance in 62 of 98 clinically high-risk cases (63%). Among these, 25 (40%) retained at least one adverse pathological feature (pT4b and/or pN +), thus confirming eligibility for neoadjuvant chemotherapy (NAC) despite inaccurate clinical staging. Conversely, 37 of 98 patients (38%) classified as high risk on CT had no corresponding pathological risk factors, representing potential overtreatment. One of four tumours (25%) staged as cT4bN + demonstrated no adverse pathological features.

Among the 37 tumours staged as clinically low risk (cT1–4aN0), 29 (78%) were confirmed as low-risk pathologically (Table 7.).

**Table 7:**
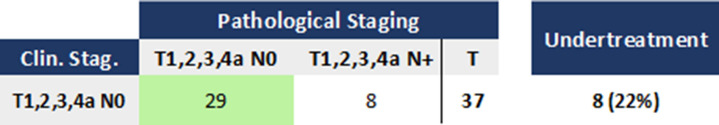
Assessment of eligibility for adjuvant chemotherapy and, consequently, for neoadjuvant chemotherapy in tumours clinically considered low risk for metastasis

Eight (22%) cases harboured at least one adverse feature, cases that would have benefited from NAC but not eligible as per CT staging.

## Discussion

Although endorsed by the NCCN guidelines, NAC has not yet become standard practice for locally advanced colon cancer, partly due to concerns about overtreatment when clinical staging relies solely on CT [23]. In real-life practice, CT interpretation is often challenging and subject to variability, which can potentially lead to misclassification of tumour stage and suboptimal treatment planning.

In this multicentre real-life cohort, CT showed limited performance in assessing both the depth of tumour invasion and locoregional lymph node status in right-sided colon cancer. Sensitivity for detecting pT4b tumours was reasonable (57%). In comparison, specificity was high (97%), reflecting a tendency toward understaging of T status. Conversely, for nodal involvement, sensitivity was high (86%) but specificity was modest (46%), resulting in frequent false positives. Overall, 38% of clinically high-risk tumours would have received unnecessary NAC, while 22% of clinically low-risk cases harboured adverse pathological features that would have justified neoadjuvant or adjuvant therapy.

These findings are broadly consistent with those of previous reports. Hernández et al. (2023) reported a sensitivity of 57% for T4 and 64% for N +, with frequent understaging (43% for T and 38% for N) and overstaging (36% for N) [14]. Nerad et al. (2016) found CT sensitivity of 90% and specificity of 69% for identifying pT3/4 disease, and 71% and 67%, respectively, for nodal status [24]. Fernández et al. (2019), in the only study focusing specifically on right-sided colon cancer, observed sensitivity and specificity of 57% and 76% for pT3/4, and 47% and 71% for pN +, respectively [7]. In contrast, earlier studies that included both right- and left-sided tumours have shown slightly higher accuracy rates, likely due to anatomical and biological differences between tumour sites [7, 14, 24]. Collectively, these data emphasise that CT tends to overestimate nodal involvement while underestimating T4b extension, particularly in right-sided tumours. These results should be interpreted in the context of a T4b classification that does not incorporate detailed characterization of retroperitoneal margin involvement. Accordingly, the accuracy of CT imaging reported in this study reflects performance in identifying T4b disease as currently defined in routine practice, rather than in predicting specific patterns of retroperitoneal margin contact, threatened margins, or true margin infiltration. Technical limitations – including reduced soft-tissue contrast, colonic peristalsis, and difficulty distinguishing tumour infiltration from inflammatory or desmoplastic reaction – may explain the reduced diagnostic performance [25–27].

Importantly, this study reflects real-world clinical conditions, where multiple radiologists interpreted CT scans according to local practice rather than being centrally reviewed. This mirrors everyday clinical decision-making, in which staging variability is inevitable. The results underline that CT-based risk stratification in localised colon cancer can be misleading, with meaningful implications for NAC selection. Overstaging may expose patients to unnecessary chemotherapy-related toxicity, surgical delay, and impaired fitness for resection, while understaging may result in missed opportunities for early systemic control of micrometastatic disease.

Alternative imaging modalities, including MRI, CT colonography, and PET, have been investigated to improve locoregional staging. MRI provides superior soft-tissue contrast and can better delineate T4 disease; however, its performance in nodal staging remains comparable to that of CT [7, 14, 24, 28]. CT colonography offers limited additional benefit and requires prior bowel preparation [29, 30], while PET lacks adequate spatial resolution for T and N assessment [29]. Therefore, CT remains the most practical modality for preoperative staging, though its diagnostic limitations must be acknowledged when considering NAC eligibility.

Modern imaging technologies, including three-dimensional (3D) reconstruction and artificial intelligence (AI)-based segmentation, can complement conventional CT and improve clinical staging and surgical planning for right-sided colon cancers. By enabling more precise characterization of tumour morphology, depth of infiltration, margin status, and spatial relationships with adjacent structures—including vasculature, fascia, and mesenteric planes—these tools enhance preoperative staging accuracy and risk stratification. This may optimize patient selection for neoadjuvant chemotherapy and support individualized surgical strategies, potentially reducing treatment-related complications and improving prognosis. Nevertheless, standardised protocols and prospective validation are required to ensure reproducibility and integration into routine clinical practice [31, 32].

This study comes with some limitations. Its retrospective design and modest sample size may limit generalizability. Incomplete clinical records restricted the evaluation of postoperative treatments and long-term outcomes, and a proportion of patients lacked complete nodal staging, limiting combined T and N analyses. Moreover, radiological data did not include parameters such as the presence of extramural vascular invasion (EMVI) and the presence of tumour deposits, which are integrated into the CT-TDV system recently proposed [33]. Eligibility for neoadjuvant chemotherapy in our analyses was based solely on T4b and N + status, without including high-risk T3 tumours, which may have underestimated the potential clinical impact of CT-based staging in identifying all high-risk patients. In addition, the number of cT4b cases was 9 and of pT4b cases of 7, the size of this category of patients limits the generalizability of our results. In this real-world cohort, detailed radiological descriptors of the retroperitoneal margin, as contact with the retroperitoneal fascia or the presence of threatened margins, were not systematically reported and therefore could not be analysed as independent variables. Consequently, cases were classified according to the binary pT4b definition. This represents a limitation of the study when interpreting the ability of preoperative CT imaging to predict retroperitoneal margin risk. At the same time, this approach reflects common daily reporting practices, which may themselves contribute to the challenges of accurately integrating margin-specific risk stratification into preoperative decision-making.

CT remains the gold-standard imaging modality for initial staging; however, its variable performance in detecting high-risk features leads to an unavoidable risk of both overstaging and undertreatment. Decisions regarding NAC should therefore be made cautiously and always within a multidisciplinary context. Future studies should assess the integration of refined radiological parameters, such as EMVI, tumour deposits, retroperitoneal margins threatening or involvement, and explore the potential of combined imaging approaches – particularly CT and MRI – and emerging imaging technologies, as 3D reconstructions and AI-based segmentation, to enhance the precision of preoperative staging and optimise patient selection for neoadjuvant strategies.

## Data Availability

The dataset is not publicly available but may be requested to the corresponding author upon reasonable request.

## References

[1] Argilés G, Tabernero J, Labianca R, Hochhauser D, Salazar R, Iveson T, Laurent-Puig P, Quirke P, Yoshino T, Taieb J, Martinelli E, Arnold D (2020) Localised colon cancer: ESMO clinical practice guidelines for diagnosis, treatment and follow-up†. Ann Oncol. 10.1016/j.annonc.2020.06.022 32702383 10.1016/j.annonc.2020.06.022

[2] Benson AB, Venook AP, Al-Hawary MM, Arain MA, Chen YJ, Ciombor KK, Cohen S, Cooper HS, Deming D, Farkas L, Garrido-Laguna I, Grem JL, Gunn A, Hecht JR, Hoffe S, Hubbard J, Hunt S, Johung KL, Kirilcuk N, Krishnamurthi S, Messersmith WA, Meyerhardt J, Miller ED, Mulcahy MF, Nurkin S, Overman MJ, Parikh A, Patel H, Pedersen K, Saltz L, Schneider C, Shibata D, Skibber JM, Sofocleous CT, Stoffel EM, Stotsky-Himelfarb E, Willett CG, Gregory KM, Gurski LA (2021) Colon cancer, version 2.2021, NCCN clinical practice guidelines in oncology. J Natl Compr Canc Netw. 10.6004/jnccn.2021.001234902826

[3] Hu Z, Ding J, Ma Z, Sun R, Seoane JA, Scott Shaffer J, Suarez CJ, Berghoff AS, Cremolini C, Falcone A, Loupakis F, Birner P, Preusser M, Lenz HJ, Curtis C (2019) Quantitative evidence for early metastatic seeding in colorectal cancer. Nat Genet. 10.1038/s41588-019-0423-x 31209394 10.1038/s41588-019-0423-x PMC6982526

[4] Agbamu DA, Day N, Walsh CJ, Hendrickse CW, Langman G, Pallan A, Lowe A, Ostrowski J, Steward M, Callaway M, Falk S, Thomas MG, Wong N, Hartley J, MacDonald AW, Blunt D, Cohen P, Dawson P, Lowdell C P, …, Lees N (2012) Feasibility of preoperative chemotherapy for locally advanced, operable colon cancer: The pilot phase of a randomised controlled trial. Lancet Oncol 13(11). 10.1016/S1470-2045(12)70348-0 10.1016/S1470-2045(12)70348-0 PMC3488188 23017669

[5] Body A, Prenen H, Latham S, Lam M, Tipping-Smith S, Raghunath A, Segelov E (2021) The role of neoadjuvant chemotherapy in locally advanced colon cancer. Cancer Manag Res 13:2567–2579. 10.2147/CMAR.S262870 33762848 10.2147/CMAR.S262870 PMC7982559

[6] Dighe S, Swift I, Magill L, Handley K, Gray R, Quirke P, Morton D, Seymour M, Warren B, Brown G (2012) Accuracy of radiological staging in identifying high-risk colon cancer patients suitable for neoadjuvant chemotherapy: a multicentre experience. Colorectal Dis. 10.1111/j.1463-1318.2011.02638.x 21689323 10.1111/j.1463-1318.2011.02638.x

[7] Fernandez LM, Parlade AJ, Wasser EJ, Dasilva G, De Azevedo RU, Ortega CD, Perez RO, Habr-Gama A, Berho M, Wexner SD (2019) How reliable is CT scan in staging right colon cancer? Dis Colon Rectum. 10.1097/DCR.0000000000001387 30870227 10.1097/DCR.0000000000001387

[8] Gosavi R, Chia C, Michael M, Heriot AG, Warrier SK, Kong JC (2021) Neoadjuvant chemotherapy in locally advanced colon cancer: a systematic review and meta-analysis. Int J Colorectal Dis. 10.1007/s00384-021-03945-3 33945007 10.1007/s00384-021-03945-3

[9] Smith NJ, Bees N, Barbachano Y, Norman AR, Swift RI, Brown G (2007) Preoperative computed tomography staging of nonmetastatic colon cancer predicts outcome: implications for clinical trials. Br J Cancer. 10.1038/sj.bjc.6603646 17353925 10.1038/sj.bjc.6603646 PMC2360118

[10] Morton D, Seymour M, Magill L, Handley K, Glasbey J, Glimelius B, Palmer A, Seligmann J, Laurberg S, Murakami K, West N, Quirke P, Gray R (2023) Preoperative chemotherapy for operable colon cancer: mature results of an international randomized controlled trial. J Clin Oncol. 10.1200/JCO.22.00046 36657089 10.1200/JCO.22.00046 PMC10022855

[11] Scott N, Jamali A, Verbeke C, Ambrose NS, Botterill ID, Jayne DG (2007) Retroperitoneal margin involvement by adenocarcinoma of the caecum and ascending colon: what does it mean? Colorectal Dis 10(3):289–293. 10.1111/j.1463-1318.2007.01365.x 10.1111/j.1463-1318.2007.01365.x 17764533

[12] Shkurti J, Van Den Berg K, Tissier RLM, Van Der Mierden S, Lahaye MJ, Beets-Tan RGH, Nederend J (2025) Diagnostic accuracy of CT for identifying high-risk colon cancer: a systematic review and meta-analysis. Eur Radiol. 10.1007/s00330-025-11844-2 40819156 10.1007/s00330-025-11844-2 PMC12953287

[13] Van Den Berg K, Van Hellemond IEG, Willems JMWE, Burger JWA, Rutten HJT, Creemers GJ (2024) Neoadjuvant chemotherapy in locally advanced colon cancer: a systematic review with proportional meta-analysis. Eur J Surg Oncol 51(3):109560. 10.1016/j.ejso.2024.109560 39869958 10.1016/j.ejso.2024.109560

[14] Nerad E, Lahaye MJ, Maas M, Nelemans P, Bakers FCH, Beets GL, Beets-Tan RGH (2016) Diagnostic accuracy of CT for local staging of colon cancer: a systematic review and meta-analysis. AJR Am J Roentgenol. 10.2214/AJR.15.15785 27490941 10.2214/AJR.15.15785

[15] Ingraham AM, Cohen ME, Bilimoria KY, Feinglass JM, Richards KE, Hall BL, Ko CY (2010) Comparison of hospital performance in nonemergency versus emergency colorectal operations at 142 hospitals. J Am Coll Surg. 10.1016/j.jamcollsurg.2009.10.016 20113935 10.1016/j.jamcollsurg.2009.10.016

[16] Baran B, Mert Ozupek N, Yerli Tetik N, Acar E, Bekcioglu O, Baskin Y (2018) Difference between left-sided and right-sided colorectal cancer: a focused review of literature. Gastroenterol Res. 10.14740/gr1062w 10.14740/gr1062w PMC6089587 30116425

[17] Idrissi MB, El Bouhaddouti H, Mouaqit O, Ousadden A, Ait Taleb K, Benjelloun EB (2023) Left-Sided Colon Cancer and Right-Sided Colon Cancer: Are They the Same Cancer or Two Different Entities? Cureus 15(4). 10.7759/cureus.37563 10.7759/cureus.37563 PMC10183151 37193477

[18] Iacopetta B (2002) Are there two sides to colorectal cancer? Int J Cancer. 10.1002/ijc.10635 12216066 10.1002/ijc.10635

[19] Stintzing S, Tejpar S, Gibbs P, Thiebach L, Lenz HJ (2017) Understanding the role of primary tumour localisation in colorectal cancer treatment and outcomes. Eur J Cancer. 10.1016/j.ejca.2017.07.016 28787661 10.1016/j.ejca.2017.07.016 PMC7505124

[20] Meguid RA, Slidell MB, Wolfgang CL, Chang DC, Ahuja N (2008) Is there a difference in survival between right- versus left-sided colon cancers? Ann Surg Oncol. 10.1245/s10434-008-0015-y 18622647 10.1245/s10434-008-0015-y PMC3072702

[21] Sasaki K, Andreatos N, Margonis GA, He J, Weiss M, Johnston F, Wolfgang C, Antoniou E, Pikoulis E, Pawlik TM (2016) The prognostic implications of primary colorectal tumor location on recurrence and overall survival in patients undergoing resection for colorectal liver metastasis. J Surg Oncol. 10.1002/jso.24425 27792291 10.1002/jso.24425

[22] Kishiki T, Kuchta K, Matsuoka H, Kojima K, Asou N, Beniya A, Yamauchi S, Sugihara K, Masaki T (2019) The impact of tumor location on the biological and oncological differences of colon cancer: multi-institutional propensity score-matched study. Am J Surg. 10.1016/j.amjsurg.2018.07.005 30384969 10.1016/j.amjsurg.2018.07.005

[23] Liu F, Tong T, Huang D, Yuan W, Li D, Lin J, Cai S, Xu Y, Chen W, Sun Y, Zhuang J (2019) Capeox perioperative chemotherapy versus postoperative chemotherapy for locally advanced resectable colon cancer: protocol for a two-period randomised controlled phase III trial. BMJ Open. 10.1136/bmjopen-2017-017637 30700474 10.1136/bmjopen-2017-017637 PMC6352769

[24] del García Álamo Hernández Y, Cano-Valderrama Ó, Cerdán-Santacruz C, Pereira Pérez F, Aldrey Cao I, Núñez Fernández S, Álvarez Sarrado E, Obregón Reina R, Dujovne Lindenbaum P, Taboada Ameneiro M, Ambrona Zafra D, Pérez Farré S, Pascual Damieta M, Frago Montanuy R, Flor Lorente B, Biondo S (2023) Diagnostic accuracy of abdominal CT for locally advanced colon tumors: can we really entrust certain decisions to the reliability of CT? J Clin Med. 10.3390/jcm12216764 10.3390/jcm12216764 PMC10648183 37959229

[25] Leufkens AM, Van Den MAAJ, Van Leeuwen MS, Siersema PD (2011) Diagnostic accuracy of computed tomography for colon cancer staging: a systematic review. Scand J Gastroenterol 46(7–8). 10.3109/00365521.2011.574732 10.3109/00365521.2011.574732 21504379

[26] Rollvén E, Holm T, Glimelius B, Lörinc E, Blomqvist L (2013) Potentials of high resolution magnetic resonance imaging versus computed tomography for preoperative local staging of colon cancer. Acta Radiol. 10.1177/0284185113484018 23550186 10.1177/0284185113484018

[27] van de Weerd S, Hong E, van den Berg I, Wijlemans JW, van Vooren J, Prins MW, Wessels FJ, Heeres BC, Roberti S, Nederend J, van Krieken JHJM, Roodhart JML, Beets-Tan RGH, Medema JP (2022) Accurate staging of non-metastatic colon cancer with CT: the importance of training and practice for experienced radiologists and analysis of incorrectly staged cases. Abdom Radiol. 10.1007/s00261-022-03573-7 10.1007/s00261-022-03573-7 PMC9463303 35798962

[28] Nerad E, Lambregts DMJ, Kersten ELJ, Maas M, Bakers FCH, Van Den Bosch HCM, Grabsch HI, Beets-Tan RGH, Lahaye MJ (2017) MRI for local staging of colon cancer: can MRI become the optimal staging modality for patients with colon cancer? Dis Colon Rectum. 10.1097/DCR.0000000000000794 28267005 10.1097/DCR.0000000000000794

[29] Kijima S, Sasaki T, Nagata K, Utano K, Lefor AT, Sugimot H (2014) Preoperative evaluation of colorectal cancer using CT colonography, MRI, and PET/CT. World J Gastroenterol 20(45). 10.3748/wjg.v20.i45.16964 10.3748/wjg.v20.i45.16964 PMC4258565 25493009

[30] Maupoey Ibáñez J, Pàmies Guilabert J, Frasson M, Boscà Robledo A, Giner Segura F, García-Granero Ximénez E (2019) Accuracy of CT colonography in the preoperative staging of colon cancer: a prospective study of 217 patients. Colorectal Dis. 10.1111/codi.14724 31161677 10.1111/codi.14724

[31] Jerí-McFarlane S, García-Granero Á, Martínez-Ortega MA, Amengual-Antich I, Robayo ÁR, Gamundí-Cuesta M, González-Argenté FX (2025) Tailored-surgery for locally advanced colon cancer based on 3D mathematical reconstruction surgical planner: observational comparative non-randomized study. Eur J Surg Oncol 51(2):109584. 10.1016/j.ejso.2025.109584 39808969 10.1016/j.ejso.2025.109584

[32] Quero G, Mascagni P, Kolbinger FR, Fiorillo C, De Sio D, Longo F, Schena CA, Laterza V, Rosa F, Menghi R, Papa V, Tondolo V, Cina C, Distler M, Weitz J, Speidel S, Padoy N, Alfieri S (2022) Artificial intelligence in colorectal cancer surgery: present and future perspectives. Cancers 14(15):3803. 10.3390/cancers14153803 35954466 10.3390/cancers14153803 PMC9367568

[33] Hodges N, Duxbury O, Corr A, Cho SH, Miskovic D, Brown G (2023) Inter-rater agreement between radiologists using the novel CT-TDV (T3c+; tumour deposits; EMVI) system in patients with potentially curable right colon cancer. Br J Radiol 96(1146):20220682. 10.1259/bjr.20220682 37000465 10.1259/bjr.20220682 PMC10230396

